# ‘A Moment for Me’: The Role of Respite in Sustaining Mental Health Carers

**DOI:** 10.1111/inm.70283

**Published:** 2026-06-12

**Authors:** Zahra Almoaber, Lorna Moxham, Christopher Patterson, Elizabeth Curtis

**Affiliations:** ^1^ College of Nursing at Imam Abdulrahman University Dammam Saudi Arabia; ^2^ School of Nursing, University of Wollongong Wollongong New South Wales Australia; ^3^ School of Nursing, Faculty of Science, Medicine and Health University of Wollongong Wollongong New South Wales Australia

**Keywords:** carers, lived experience, max van Manen, mental illness, phenomenology, respite care

## Abstract

Evidence suggests respite care reduces carers' burden by providing temporary relief, reducing stress and ensuring safety. Most research focuses on carers of people with dementia or physical disabilities, leaving a gap in understanding carers of individuals with mental illness (MI). Few studies explore their specific needs, indicating a crucial area for further research. This study utilised phenomenology to examine the perception of respite for those who care for people with mental illness. An interpretative phenomenological approach guided and informed the philosophy of the study. A purposive sample of 14 caregivers who utilise respite care (*n* = 14) provided narrative data through individual semi‐structured interviews. Verbatim transcripts were analysed using van Manen's approach revealing eight key elements, four primary themes and an overarching essence of meaning. The themes include (1) feeling overwhelmed, (2) the process of obtaining respite, (3) facility suitability and (4) the need to keep going. The overarching essence of meaning was identified as “Constant care is constant.” This study presents an in‐depth analysis of the findings, supplemented by a comprehensive exploration based on established theoretical frameworks. Carers benefit from respite services by improving their mental and physical health and quality of life, but service delivery needs enhancement. Service providers should improve communication, offer flexible scheduling, and tailor services to individual needs. Training and education for respite care workers, especially in managing challenging situations and providing emotional support, are vital. Raising awareness through collaborations with healthcare providers and community organisations can better inform carers about available options.

## 1 Introduction

Mental illness (MI) ranks among the top 10 global health burdens (Mental Disorders Collaborators 2022). Currently, depression affects 5% of the global population, and anxiety 3.8%, with an estimated economic impact of about US$1 trillion annually (Sapien Labs 2023). Schizophrenia affects about 20 million people worldwide, and most individuals who die by suicide have a history of mental illness (WHO 2022). The United Kingdom's National Health Service spent £14.3 billion on mental health services in 2020–2021 (Baker 2021) while in the United States, one in five (20%) adults in 2024 reportedly had a mental health condition, costing $282 billion USD (Ward et al. 2024). In Australia, the context of this paper's study, the economic burden is similarly significant. Recent estimates indicate that mental illness costs the economy approximately AUD $60–70 billion annually, incorporating healthcare expenditure, productivity losses, and reduced quality of life (Productivity Commission 2020; Deloitte Access Economics 2022).

Recognising the impact of mental illness on well‐being, the Australian government has committed to improving mental health care (Fusar‐Poli et al. 2020). While inpatient services remain a crucial component of the system, they are also resource intensive. In 2021–2022, the average cost per bed per day was $1783, exceeding the target by $401 (Government of Western Australia East Metropolitan Health Service 2023). On a national scale, public hospital mental health admitted patient care in 2022–2023 averaged AU$1532 per patient day (AIHW 2023), underscoring the urgent need for sustainable, cost‐effective strategies in mental health service delivery.

In this context, informal carers play an essential role. Individuals with MI often live with significant disabilities, requiring ongoing support from carers (Brighton et al. 2016). About 2.65 million Australians, nearly 11% of the population, serve as informal carers, with one‐third acting as primary carers (AIHW 2021). Informal carers assist with daily activities, medication, emotional support, crisis intervention, and navigating health systems (AIHW 2021; Krysinska et al. 2022). They also provide significant economic value. In Australia, unpaid caregiving adds over AUD $130 billion annually by reducing healthcare use and delaying institutional care (Deloitte Access Economics 2022; Productivity Commission 2020).

Carers for people with MI face a broad range of physical and emotional demands, emphasising the importance of targeted support services. While formal carers are paid professionals, informal carers, usually family members or friends, offer unpaid care. These carers encounter both objective burdens, such as physical health issues, and subjective burdens, such as emotional distress (AIHW 2021). In the UK, one in four adults provides unpaid care, many of whom report compromised health and a lower quality of life compared to the general population (Carers 2021). Providing carers with respite could help ease their burden (Almoaber et al. 2024), reduce overall healthcare costs, and enhance the sustainability of the mental health system (Schofield et al. 2022).

Respite care offers temporary relief allowing carers to rest and recharge, while providing care recipients with opportunities for social engagement and continuity of care (American Psychiatric Association 2022). In Australia, formal respite options include in‐home support and residential care, designed to meet diverse needs. An integrative review found that respite care can reduce carer burden and enhance social interaction; however, limited awareness of services and previous negative experiences often constrain uptake. To maximise the benefits of respite care for carers of people with mental ill health, services must acknowledge and address the unique challenges associated with mental illness (Almoaber et al. 2024).

Despite established advantages of respite care, research has predominantly concentrated on caregivers supporting individuals with physical disabilities or age‐related conditions. Conversely, less attention has been directed toward caregivers of individuals with mental illnesses. Although a substantial body of evidence underscores the experiences, burdens, and support requirements of informal caregivers within mental health settings (Yesufu‐Udechuku et al. 2015; Harvey et al. 2016), comparatively limited research has explored their utilisation of respite services or the factors that motivate or discourage their use of such services. Addressing this gap is essential to developing services that genuinely support caregivers and optimise benefits for both the economy and the healthcare system.

### 1.1 Study Aim

To explore informal carers' perceptions and experiences of respite care in the context of supporting people with mental illness.

### 1.2 Research Design

This qualitative study investigated the lived experiences of respite care among informal carers of adults with mental illness.

### 1.3 Methodology

An interpretive phenomenological approach, informed by hermeneutics, was employed to explore the meaning of participants' experiences. van Manen's approach was chosen because it aligns well with the study's aim of exploring and interpreting the lived experiences of carers, with a focus on meaning, context, and the everyday lifeworld. This framework allowed for a more profound engagement with participants' stories, aiding in the interpretation of both explicit narratives and the deeper meanings within them.

### 1.4 Methods

Data were collected through semi‐structured interviews and analysed using van Manen's (1997, 2014) phenomenological framework to capture the essence of carers' lived experiences.

Purposive sampling selected carers who met the inclusion criteria of caring for adults over 18 with mental illness, having used respite care and speaking English. The sampling strategy enabled in‐depth insights into the phenomenon. Recruitment was conducted via organisations supporting carers, with flyers and a participant information sheet distributed by email. Interested individuals contacted the first author, provided informed consent, and scheduled an interview.

Open‐ended questions elicited detailed descriptions of participants' experiences (see Table 1). The first author conducted interviews online, via telephone, or in person, depending on participant preference. Most participants chose telephone interviews, citing convenience, geographic distance, and greater comfort when discussing personal experiences. Interviews lasted between 30 and 45 min.

**TABLE 1:** Interview questions.

Interview questions	Example probes (used where appropriate)
When you were experiencing respite, how did you spend your time?	How did this time differ from your usual routine? How did you feel during and after this time?
Are there any barriers to you accessing respite? If yes, what are they?	Can you give an example of a time you faced difficulty accessing respite? How did these challenges affect you?
In what ways does respite contribute to your own mental health?	Can you describe any changes in your wellbeing? Can you share a specific example?
How important is it for you to have access to respite?	Why do you feel it is (or is not) important? How does it impact your daily life?

*Note:* These questions served as a flexible guide. Additional probing questions were used where appropriate to explore participants' lived experiences in greater depth, consistent with a semi‐structured interview approach.

### 1.5 Ethical Consideration

Prior to data collection, the University of Wollongong granted ethical approval. At the outset, the interviewer reiterated the purpose of the study, emphasised the voluntary nature of participation, and confirmed the use of audio recording. Verbal consent was obtained and documented at the beginning of each interview.

### 1.6 Data Analysis

Data analysis in phenomenological research involves holistic and interpretive strategies that situate participants' narratives within the broader context of their lived experiences, recognising the interrelatedness of meanings across the phenomenon (Creswell and Creswell 2018). This study was guided by the interpretive phenomenological framework of van Manen (van Manen 1990), which supports a reflective and contextually grounded exploration of lived experience.

van Manen proposes six methodological activities that guide phenomenological inquiry; however, these are not intended as linear steps but as dynamic and iterative processes. Accordingly, the analysis involved continuous movement between parts and the whole, consistent with the hermeneutic circle (Gadamer 1975). This iterative engagement enabled deep interpretive reflection on participants' accounts. To enhance analytic rigour, a constant comparative approach was incorporated as a flexible analytic strategy to examine similarities and differences across meaning‐rich statements within and between transcripts. While constant comparison is traditionally associated with grounded theory (Glaser and Strauss 1967), its use in this study was adapted to support phenomenological analysis by facilitating careful comparison and refinement of experiential meanings rather than theory generation (Boeije 2002; Charmaz 2006).

The analysis involved repeated holistic readings of transcripts, highlighting significant statements, and a detailed line‐by‐line review to identify core themes. This ensured that interpretations remained grounded in participants' accounts and preserved their complexity (van Manen 1990; Finlay 2011). Reflexivity was integrated through ongoing awareness of the researcher's positionality and experience, with bracketing as a continuous practice of suspending preconceptions by revisiting verbatim accounts (Giorgi 2009). This iterative approach enhances transparency, rigour, and credibility (Table 2).

**TABLE 2:** The six steps of van Manen's interpretive phenomenological research framework.

Steps 1–6	Application to current study
“Turning to the nature of the lived experience” (van Manen 1997, 30). In this initial phase, the researcher selects a phenomenon that is of special interest while formulating a research question	The research question was: “What are the experiences of carers who utilise respite for people with mental illness? The phenomenon of interest was the lived experience of respite from the perspective of informal carers”
2 “Investigating the experience as we live it rather than as we conceptualise it” (van Manen 1997, 30). The researcher becomes immersed in the phenomenon, taking nothing for granted. The ego‐logical starting point, as van Manen terms it, is the researcher's personal experience when employing this investigative approach	In‐depth semi‐structured interviews were used to gather data, enabling a rich exploration of the phenomenon. This common qualitative method facilitated meaningful dialogue between researcher and participant, allowing for deeper insights into the lived experience of respite. In acknowledging my ego‐logical starting point, the first author drew on 7 years of experience as a mental health lecturer in Saudi Arabia. As discussed in the methodology section, this background informed my understanding and could not be entirely bracketed. Instead, it was recognised as part of the interpretive process
3 “Reflecting on the essential themes which characterise the phenomenon” (van Manen 1997, 30)	This stepconcentrated on identifying and articulating the core themes that emerged from the interviews aiming to capture the fundamental meaning of the lived experience of respite. The analysis followed the Constant Comparative Method (CCM), progressing from a holistic reading of each transcript to selective highlighting of specific statements, and finally to line‐by‐line examination. This iterative process supported the development of essential themes that reflected the shared experiences of participants
4 Describing the phenomenon through the art of writing and rewriting to bring the experience from internal to external (van Manen 1997, 30)	Thematic description and discussion aimed to make participants' feelings, thoughts, and attitudes visible through a reflective and iterative process. This approach uncovered embedded meanings and brought their experiences to the fore. Rich, detailed descriptions were constructed that conveyed interpretation while externalising an evolving understanding of their lived experiences
5 “Maintaining a strong connection and oriented relation to the phenomenon” (van Manen 1997, 33)	Intentional focus on the phenomenon of interest (experience of respite), was maintained throughout the research process. This required continuous and deliberate effort to ensure that all stages of analysis and writing stayed grounded in participants' lived experiences
6 “Balancing the research context by considering the parts and whole” (van Manen 1997, 33). The research process and writing of the text are iterative processes that are continually refined as meanings and understandings reveal themselves	The study design was continually evaluated in relation to its broader contextual purpose. This involved maintaining awareness of the whole while attending to each individual component, ensuring coherence and alignment. By balancing the parts with the overall perspective, a unified and meaningful representation of the phenomenon was presented

### 1.7 Rigour of Findings

Rigour of the study was maintained by adherence to the criteria of trustworthiness including credibility, dependability, confirmability, and transferability. Credibility, or confidence in the authenticity of findings (Holloway and Galvin 2016), was supported by prolonged engagement, detailed transcription, supervisor triangulation, and peer review (Polit and Beck 2017). Dependability, the consistency of findings, was addressed through clear documentation of data collection and analysis. Confirmability was strengthened through a reflexive journal that captured post‐interview insights and potential biases. Transferability, or relevance to similar contexts, was supported by detailed descriptions of the study's aims, methods, and findings, enabling others to access applicability.

## 2 Findings

Fourteen carers for individuals with mental illness participated in this study. All were female, most caring for adult children, but three were providing care for a partner or husband. Ages ranged from 30 to 79, with the duration of caring from 4 to 40 years (see Table 3).

**TABLE 3:** Participant demographic data.

	Caregivers name	Age group	Gender of caregiver	Care recipient	Duration of caring	Respite care	Length of respite (days)	Location
1	Marley	30–39	Female	Partner	4–5 years	Recovery camp	5	NSW
2	Grace	60–69	Female	Son	29 years	Recovery camp	5	NSW
3	Leeane	40–49	Female	Partner	5 years	Recovery camp	5	NSW
4	Judy	60–69	Female	Son	20 years	Recovery camp	5	NSW
5	Belinda	60–69	Female	Daughter	14 years	Recovery camp	5	NSW
6	Jana	50–59	Female	Son	15 years	Carer NSW	2	NSW
7	Anne	70–79	Female	Daughter	40 years	Recovery camp	5	VIC
8	Lea	40–49	Female	Son	14 years	Stride	1day	NSW
9	Mag Eli	50–59	Female	Son	8 years	One Door MH	2	NSW
10	Jenni	50–59	Female	Son	15 years	Stride	1day	NSW
11	Anne B	60–59	Female	Daughter	9 years	Recovery camp	5	NSW
12	Penny	70–79	Female	Son	23 years	Recovery Camp	5	NSW
13	Deborah	50–59	Female	Son	10 years	Recovery camp	5	NSW
14	Kim	60–69	Female	Husband	10 years	Recovery camp	5	NSW

**FIGURE 1:**
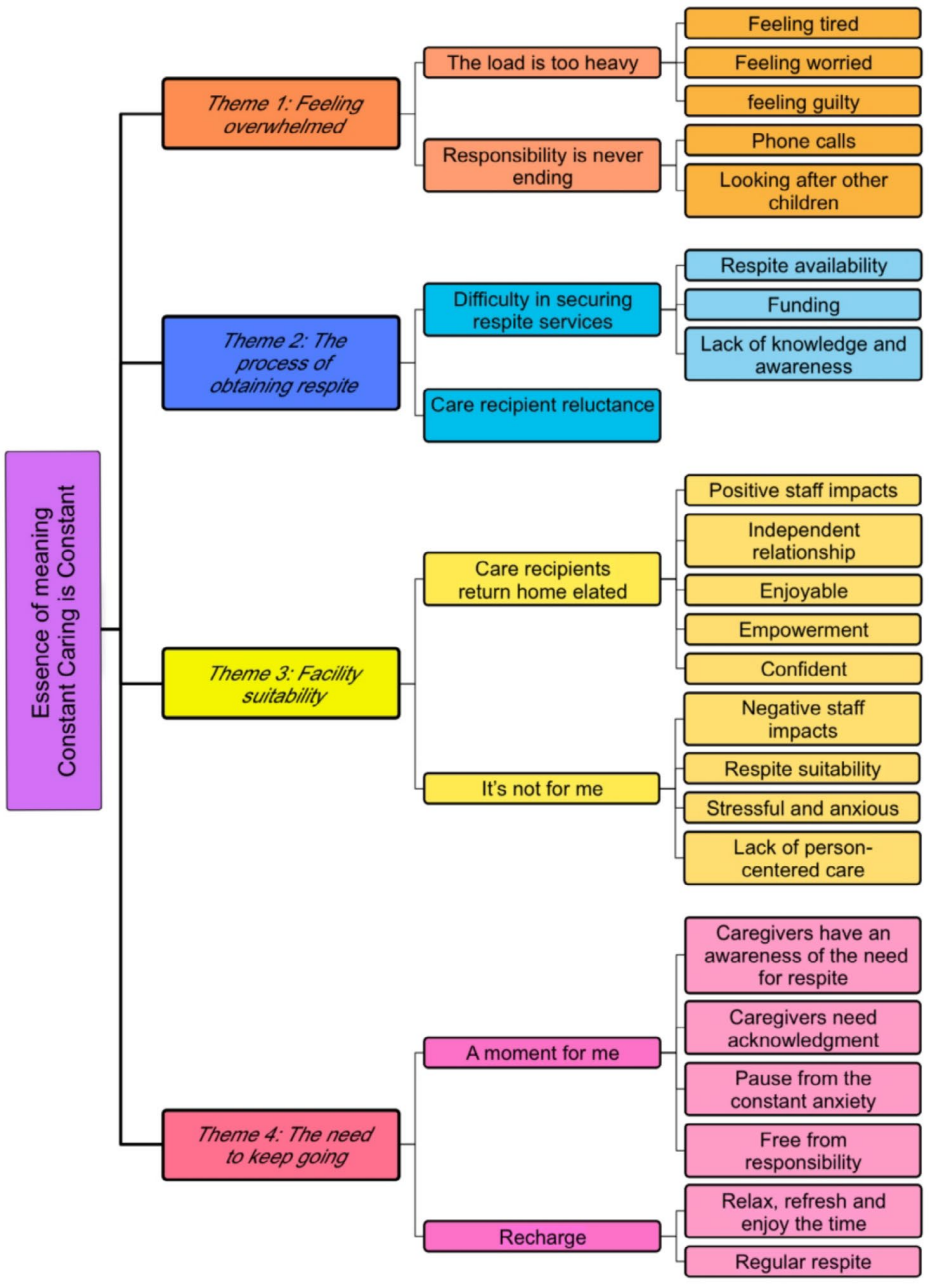
The relationship between essence of meaning, themes and elements.

Eight elements were organised into four interrelated themes, which together represent the essence of meaning: *Constant Caring is Constant*. Figure 1 illustrates the relationship between elements, sub‐elements and their respective themes, the interconnected nature of the four themes, and their collective role in conveying the essence of meaning.

### 2.1 Theme 1: Feeling Overwhelmed

Carers described the emotional and psychological burden of supporting someone with mental illness as overwhelming. Feeling overwhelmed arose from the unpredictability of the illness, constant demands, emotional distress, and lack of support. Reported symptoms included anxiety, persistent worry, exhaustion, irritability, and feelings of helplessness or hopelessness.

This theme is informed by two elements: (a) The load is too heavy and (b) Responsibility is never‐ending.

#### 2.1.1 The Load Is Too Heavy

Carers described feeling overwhelmed by the demands of their roles, which affected their physical and emotional well‐being. Balancing caregiving with other responsibilities often led to exhaustion, isolation, and limited opportunity for self‐care. Many described the burden as too heavy to carry alone, with persistent feelings of tiredness, worry, and guilt.

Most carers reported frequent exhaustion, not only from daily household tasks, but from the emotional labour of monitoring well‐being, managing mood, and supporting personal care needs. Caregiving was said to be relentless.

I'm drained all the time. You know, I used to do everything in the house before the NDIS came. I used to cook, clean, shower, make beds, dress him, and do the shopping. All the time I am tired (Marley).

Carers also expressed guilt when taking time to rest, feeling they lacked permission to step away. Jana described her inability to enjoy respite care, overwhelmed by concern for her son's distress. Jana's emphasis is on the emotional and cognitive burden of attempting to disengage through respite yet remaining psychologically and relationally “on duty.”

“I feel a bit guilty because I know he finds it very stressful to be away overnight. I used respite care twice because it wasn't worth the stress that I was putting my son under, and I didn't enjoy it. You know, I can't relax, I said, carer knowing that he's being adversely impacted”. (Jana)

#### 2.1.2 Responsibility Is Never‐Ending

Although carers may view respite as a break from caregiving, true rest is often elusive. Responsibilities persist in various forms, including managing phone calls, completing household tasks, and caring for other children. Even during respite, carers remain engaged. Many continue to monitor the well‐being of their loved one by maintaining contact, ensuring medications are taken, and offering reassurance. Jana described how caregiving extends beyond mere physical presence.

I still text just to check in with him and just a reminder about medication times and just to reassure him that he is OK. He would also text me as well a few times a day … so I'm still 24/7 no matter what, I'm doing (Jana).

For carers with other children, responsibilities multiply, further limiting the opportunity for genuine respite. Belinda explained how ongoing caregiving demands prevent meaningful rest.

Belinda explained that caring for additional children prevents them from benefiting from respite care.

“I have a responsibility that never ends; I need to take care of other children… The respite would be better if we could have a plan for rest. I can't plan a holiday because I have other kids to care for …” *(Belinda)* .

### 2.2 Theme 2: The Process of Obtaining Respite

Respite may involve the use of professional services, community support, or assistance from friends and family. However, access to respite depends on individual circumstances and available resources. This is illustrated through the two elements that inform this theme: (a) It is just so very hard and (b) Care recipients' reluctance.

#### 2.2.1 Difficulty in Securing Respite Services

Securing respite services was often challenging, shaped by three key barriers: availability, funding, and lack of knowledge and awareness.

Carers begin by assessing their needs, often exploring options through professional services, community programs, and/or care facilities. However, limited availability can restrict choice, leading carers to repeatedly use the same services and potentially miss more suitable alternatives. Grace noted a lack of awareness about options.

There was a little first one he went to where he wanted to be around Wagga. I don't know what they call it, and I only know the one there, and he doesn't go there anymore. There is one in Richmond (Jody).

Financial stress is another barrier. Many carers struggle to take breaks due to the cost of respite care. Without adequate financial support, basic needs may go unmet, compounding emotional strain and reducing access to rest. Anne B describes the impact of financial hardship.

…the cost needs to be affordable, which is the problem. Financial stress becomes an issue when you are a carer and are already distressed. It's essential for[respite] to be affordable as I've encountered financial problems before. Sometimes, even filling up the car with petrol can be a struggle. I can only afford to put in enough for half a tank (Anne B).

Carers may not fully understand the value of respite or how to seek appropriate support. Mag Eli advocated for early mental health education as a way to build awareness and encourage help‐seeking.

I just thought we needed to have mental health education in high I said I couldn't understand what my son was going through, and I wanted to help him I made friends with them as well, and they were the ones pushing me to go to ARAFMI … the school education would have helped us (Mag Eli).

#### 2.2.2 Care Recipient Reluctance to Respite Services

Care recipients' experiences are discussed within this theme because they directly influence carers' decisions and emotional responses, and therefore affect carers' ability and willingness to use respite services.

This theme refers to situations in which respite services are accessed by the person living with mental illness, and where their reluctance or distress limits carers' ability to use respite. Carers described resistance from care recipients due to discomfort with unfamiliar providers, concerns about service quality, or perceptions that accepting respite signified vulnerability. Consequently, respite was often declined, restricting carers' access to support. For example, Jana explained that her son's preference for one‐to‐one support over group‐based respite prevented her from using available services.

… we've offered him many groups and our teams over the years and he always declines umm so he prefers just to be one‐on‐one, …… The other barrier is that my son is not consenting often to the referral, so I can't go ahead with it because it's about him (Jana).

I feel a bit guilty because I know he finds it very stressful to be away overnight. I used respite care twice because it wasn't worth the stress that I was putting my son under, and I didn't enjoy it. You know, I can't relax, I said, carer knowing that he's being adversely impacted. (Jana)

I still text just to check in with him and just a reminder about medication times and just to reassure him that he is OK. He would also text me as well a few times a day … so I'm still 24/7 no matter what, I'm doing (Jana).

### 2.3 Theme 3: Facility Suitability

Participants highlighted that the suitability of a respite facility significantly shapes both the care recipient's experience and the carers' well‐being. A positive experience can result in benefits for the carer. Conversely, unsuitable facilities may cause distress, anxiety, or reluctance to return. Factors such as impersonal care and poor amenities can lead to dissatisfaction and undermine the intended benefits of respite.

This theme is informed by two elements: (a) Care recipients return home elated and (b) It's not for me.

#### 2.3.1 Care Recipient Returns Home Elated

A positive respite experience can leave care recipients feeling elated, supported and more confident. This element comprises five sub‐elements: (1) Positive staff impacts, (2) Independent relationship, (3) Enjoyable, (4) Empowerment, and (5) Confident.

Competent, empathic staff enhance the quality of care and contribute to both care recipient satisfaction and carer reassurance. Effective communication, medication management, and behaviour support are central to building trust and reducing carer stress. As Belinda stated:

“They [respite staff] would need to be able to manage medication, so they would need, you know, nursing experience, and they would need to be able to manage behaviours of concern” (Belinda).

Open communication further supports well‐being and reinforces relationships:

Communication between staff and care recipients is a big issue that is not only about the carers but is also about care recipients (Penny).

Many carers reported that their loved ones returned from respite more socially engaged, confident and emotionally refreshed. Respite allows care recipients to form friendships, engage in meaningful activities and develop independence. Deborah shared:

I think respite is important for me, but it is also important for my son too, to have a break. My son loves it because he meets other people and has friends, and has a break from me too. It is nice for him to get away; it is a holiday for him, and it is really good. He had a very good experience. He comes back happier, refreshes, and makes life easier. He is excited to have respite, it's really good for him (Deborah).

Marley noted the impact of social and recreational engagement:

He's brighter, and he's just happier. He made friends, he just wants to bake cakes (Marley).

Respite also fosters empowerment by allowing care recipients to exercise autonomy and regain a sense of control. Mag Eli emphasised the importance of trust in this process:

I've taken a risk because I could have found him dead when I came back, but I'd never left him if I knew it was in the middle of a crisis at the top left, of course, but I was just, you know, trusting that it wouldn't disappear or take off (Mag Eli).

Respite care can also improve self‐confidence, as seen in Grace's son, who returned more socially assured after participating in ‘Recovery Camp’. Previously, he was shy and avoided groups.

a little bit more confident because he's very shy, he doesn't like crowds of people before, but he just seemed really happy and was happy when he came home (Grace).

#### 2.3.2 It's not for Me

This element comprises four sub‐elements: (1) Negative staff impacts, (2) respite suitability, (3) stress and anxiety, and (4) person‐centred care.

Poorly trained or disengaged staff can create negative experiences, leading to frustration, anxiety, and a sense of being misunderstood. These interactions can erode trust in the facility and discourage future use.

Penny described how untrained staff, lacking basic interpersonal engagement, contributed to a stressful environment:

The staff is not trained to deal with people with mental illness. They sit on the couch on their phones. There is no interaction, and they do not get paid to sit on their phone. … The client is struggling with whether he has any kind of disability and gets some and doesn't interact, which creates a language barrier. A lot of staff have aggressive natures and don't help anybody. They should show they are going to assess not being decentred and tell them what to do all the time, and they want to engage (Penny).

When respite facilities are poorly matched to the needs to the care recipient, the result can be stress and reluctance to return. Jana shared how her son found respite particularly stressful due to staff being ill‐equipped to support him:

It is very stressful for him to stay too long or overnight, the staff was not cooperative, and well trained to deal with him (Jana).

These experiences highlight the importance of person‐centred care, with its emphasis on tailoring care to individual needs and preferences. Carers emphasised that the success of respite depends on environments that are welcoming, safe, and suited to the unique requirements of both the care recipient and their family.

### 2.4 Theme 4: The Need to Keep Going

Carers provide essential support and taking time for themselves is vital to sustaining their role. By restoring their physical, emotional, and mental energy, carers maintain their capacity to provide quality care. Findings emphasise the importance of supporting carers to prioritise their own well‐being, which is crucial for the longevity of their caregiving role.

This theme is informed by two elements: (1) A moment for me and (2) Recharge.

#### 2.4.1 A Moment for Me

Respite provides carers with opportunities to engage in self‐care, pursue personal interests, or rest. Participants described how time away from caregiving helped alleviate stress and manage the demands of their role. Jinne highlighted the cumulative toll of continuous caregiving without breaks and described how time away enabled a sense of reset and improved functioning upon return:

*Be able to relax and get yourself back on track so you can be a better carer. Because the more stressed we are, we do the worst job, the more we can sort of get a break and be able to reset a little bit. I think that you know we're much better carers for that*. (Jinne)

Carers also spoke about the importance of feeling acknowledged and supported when accessing respite. Jana described the need for affordable respite options and emphasised that trust in the quality of care was essential for her to feel able to disengage during a break:

*Let them acknowledge and value carers and that we do need regular breaks. But it needs to be affordable, I can't afford to go away. The quality of care for a loved one needs to be high quality so that we can truly relax and trust everything's going to be normal, not worrying about the quality of respite for our loved ones. We're not going to get any benefit out of the break*. (Jana)

Participants also described respite as providing temporary relief from constant vigilance and emotional strain. Lea explained how even short periods of respite reduced ongoing anxiety:

*I guess it just puts a pause on all these stresses and worries instead of worrying 24/7, you know, the moment I open my eyes is kind of like a break from the constant anxiety*. (Lea)

Similarly, Grace reflected on the calming effect of time away from caregiving responsibilities, particularly the absence of routine domestic tasks:

*I just have to not worry about whether I have to cook. If I can, just little things. I just don't. It's because I like that it's quiet, and I don't have to worry about cooking, cleaning, making a noise, or doing anything. Yeah, that was just really very calming*. (Grace)

#### 2.4.2 Recharge

Respite care plays a vital role in enabling carers to restore their emotional, physical and mental reserves. These breaks provide an opportunity to temporarily step back from intensive caregiving responsibilities, returning renewed and better equipped to manage challenges with empathy, patience and resilience. Recognising the necessity of recharging supports the long‐term sustainability of caregiving and reinforces the importance of the carers' own well‐being. Maintaining this balance is essential not only for the health of the carers but also for the continuity of high‐quality care for their loved ones.

Grace articulated the profound benefits of respite when she said:

It's just the two of us here this time break away from each other. It's really important for me because I am not just mum but also her carer. Just for that week, I wasn't his carer. I was just mum, yeah, and that's really nice, and it's really important that my son had a break away too. He had something to look forward to, and he had people relying on them that he had to be very committed, and that's made him, I think, a lot mentally stronger. … it's really really important that we recharge our batteries (Grace).

This account illustrates how respite care benefits both carer and care recipients by promoting personal well‐being, encouraging independence, and fostering mutual growth. Supporting carers to take such breaks ultimately strengthens their ability to continue providing essential care over time.

## 3 Discussion

This discussion integrates the findings of the present interpretive phenomenological study that explored the lived experiences of carers of people with mental illness who utilise respite care. Four overarching themes were identified from the semi‐structured interviews: (1) *Feeling overwhelmed*, (2) *The process of obtaining respite care*, (3) *Facility suitability*, and (4) *The need to keep going*. The core meaning that originated from these findings was “Constant Caring is Constant,” supported by existing literature on caregiving and respite care in the context of mental illness. By comparing carers' lived experiences with prior empirical research, the analysis highlights areas of convergence and divergence and extends existing knowledge by shedding light on how carers make sense of respite beyond service availability or outcomes alone.

Meaning making is central to phenomenological inquiry, requiring critical reflection to move beyond surface descriptions toward deeper experiential understanding (Hidayati et al. 2020). In this study, carers' narratives revealed that caregiving for a person with mental illness is experienced as a sustained, unwavering commitment (Constant Caring is Constant) rather than an intermittent role. This finding aligns with Jeon et al. (2006), who describe caregiving as a continual presence that becomes a defining feature of carers' lives. However, this study extends the literature by demonstrating how this enduring commitment shapes carers' emotional responses to respite, including feelings of guilt, responsibility, and vigilance, even during periods intended for relief.

In line with previous research, participants in this study reported high levels of stress and being overwhelmed (Aung et al. 2021). Participants identified multiple factors contributing to this, including concerns about the quality of care during respite, anxiety about entrusting others with their loved ones, and a lack of systemic support to meet their broader needs as carers. While Wakefield (2020) similarly highlights that stress often persists despite the availability of respite, this study deepens that understanding by showing how carers' stress is closely linked to relational trust and moral responsibility, not just access to services. This finding challenges the idea that respite automatically reduces caregiver burden and highlights the importance of carers' personal views of their respite experiences.

The multidimensional burden described by participants reflects extensive literature documenting physical, emotional, psychological, and financial strain among carers of people with mental illness (Funk et al. 2019; Gao et al. 2023; Gusdal et al. 2024). This study reinforces these findings and shows how these burdens are exacerbated by practical barriers to accessing respite, including geographic distance, financial pressures, potential job loss, and carers' own health issues. Feelings of guilt and concern were heightened when respite facilities were hard to access or considered unsuitable, further discouraging engagement. These findings build on previous work by illustrating how structural barriers and emotional burdens interplay to create ongoing feelings of isolation, disconnection, and overwhelm (Smith 2024; Woodgate et al. 2023).

Carers' accounts highlighted the complex and often inaccessible nature of respite systems, echoing prior research that identifies limited service availability and difficulties navigating healthcare systems as significant barriers (Leocadie et al. 2018; O'Shea 2020; Funk et al. 2019). Access to respite was influenced by geographic location, socioeconomic status, insurance coverage, and the complexity of care recipients' needs. As Wicks (2023) notes, individuals facing social vulnerabilities frequently encounter systemic barriers within healthcare, underscoring the need for clearer communication and tailored support. While some carers in this study were able to navigate the system, others, particularly those managing complex or high‐intensity caregiving, found the process fragmented, inconsistent, and overwhelming, consistent with Ryvicker's (2018) findings. These results support previous calls for improved planning, coordination, and delivery of respite services, especially for individuals with complex mental health needs (Knighting et al. 2021; Sobotka et al. 2019).

Barriers to respite care increased due to limited awareness of supports and difficulty accessing timely information. Participants were unsure about eligibility, services, and referrals, aligning with Phillipson et al. (2019). Care recipients' reluctance to use formal services, common in mental health research (McPherson et al. 2018; Ogunjesa et al. 2024), left carers in a difficult position. Their need for relief conflicted with recipients' resistance, intensifying carers' emotional stress.

Facility suitability proved to be a key factor influencing carers' perceptions of respite. When respite settings were seen as responsive, safe, and meeting care recipients' needs, carers felt more reassured and more willing to use the services. Importantly, carers judged the success of respite mainly by the experiences of care recipients. Positive outcomes, especially when care recipients returned home feeling happy or fulfilled, reinforced carers' willingness to use respite again and encouraged future engagement, aligning with O'Shea et al. (2019). Conversely, negative experiences deterred further use, heightened carers' concerns, and reduced trust in services (Glynn and Mayock 2023). These findings expand the current understanding by highlighting that carers' uptake of respite depends on the quality of the care recipients' experiences and is relationally mediated, not just driven by carers' own needs.

Despite these challenges, carers in this study described using a variety of coping strategies to maintain their caregiving roles, including problem‐solving, seeking social support, and engaging in leisure activities. These strategies highlight the vital role of coping in helping carers ‘keep going’ despite ongoing demands (Epiphaniou et al. 2012). Many participants also shared personal growth over time, such as developing new skills, building resilience, and gaining a deeper understanding of their own strengths and limitations, consistent with Carter (2023). Support from family, friends, and professional services was identified as essential in building resilience by offering both emotional reassurance and practical assistance (Perera and Standen 2014).

Carers in this study viewed respite as essential, describing it as “taking a moment for me” that eased caregiving's constant anxiety. They emphasised caregiving as a marathon, with exhaustion reducing their capacity for quality care (Owoo et al. 2022). Respite was seen as vital for maintaining self, wellbeing, and long‐term care (Feng et al. 2020).

## 4 Conclusion

This study explored the lived experiences of carers for individuals with mental illness who access respite care. The core essence identified is that Constant Caring is Constant. Carers reported feeling overwhelmed, particularly by the challenge associated with accessing respite services and concerns regarding the effectiveness and suitability of facilities. Despite these difficulties, the findings highlight that carers recognise the necessity of continuing their caregiving role, often sustained through coping strategies and the critical support provided by respite care.

## 5 Contribution to Practice

This study differs from much of the existing research by focusing on how caregivers interpret the meaning of respite, rather than viewing it primarily as a service outcome or intervention. By emphasising caregivers' personal experiences, it highlights the emotional, relational, and moral aspects of respite care that are often overlooked in quantitative studies or service evaluations. These insights have practical implications for clinical practice. Mental health professionals and service providers should understand that respite is experienced not just as a break from caregiving but as a relational process involving trust, guilt, and perceived care quality. Engaging caregivers and care recipients in planning, improving communication, and ensuring consistent, responsive care can boost caregivers' confidence, increase service utilisation, and support more sustainable care arrangements.

## Funding

The authors have nothing to report.

## Conflicts of Interest

The authors declare no conflicts of interest.

## Data Availability

Research data are not shared.
